# Co-Designing a Mobile App to Improve Mental Health and Well-Being: Focus Group Study

**DOI:** 10.2196/18172

**Published:** 2021-02-26

**Authors:** Felwah Alqahtani, Andrea Winn, Rita Orji

**Affiliations:** 1 Faculty of Computer Science Dalhousie University Halifax, NS Canada; 2 Faculty of Computer Science King Khalid University Abha Saudi Arabia

**Keywords:** mental health, mobile app, focus groups, design recommendation, mobile phone

## Abstract

**Background:**

Recent advances in mobile technology have created opportunities to develop mobile apps to aid and assist people in achieving various health and wellness goals. Mental health apps hold significant potential to assist people affected by various mental health issues at any time they may need it, considering the ubiquitous nature of mobile phones. However, there is a need for research to explore and understand end users’ perceptions, needs, and concerns with respect to such technologies.

**Objective:**

The aim of this paper is to explore the opinions, perceptions, preferences, and experiences of people who have experienced some form of mental health issues based on self-diagnosis to inform the design of a next-generation mental health app that would be substantially more engaging and effective than the currently available apps to improve mental health and well-being.

**Methods:**

We conducted six focus group sessions with people who had experienced mental health issues based on self-diagnosis (average age 26.7 years, SD 23.63; 16/32, 50% male; 16/32, 50% female). We asked participants about their experiences with mental health issues and their viewpoints regarding two existing mental health apps (the Happify app and the Self-Help Anxiety Management app). Finally, participants were engaged in a design session where they each sketched a design for their ideal mental health and well-being mobile app.

**Results:**

Our findings revealed that participants used strategies to deal with their mental health issues: doing something to distract themselves from their current negative mood, using relaxation exercises and methods to relieve symptoms, interacting with others to share their issues, looking for an external source to solve their problems, and motivating themselves by repeating motivational sentences to support themselves or by following inspirational people. Moreover, regarding the design of mental health apps, participants identified that general design characteristics; *personalization of the app,* including *tracking and feedback, live support,* and *social community;* and providing *motivational content* and *relaxation exercises* are the most important features that users want in a mental health app. In contrast*, games, relaxation audio, the Google map function, personal assistance to provide suggestions, goal setting,* and *privacy preservation* were surprisingly the least requested features.

**Conclusions:**

Understanding end users’ needs and concerns about mental health apps will inform the future design of mental health apps that are useful to and used by many people.

## Introduction

### Background

Mobile health technology is considered to be a promising tool to help users engage in their health care. Specifically, the ubiquitous nature of smartphones and other handheld devices makes them ideal tools for delivering mental health interventions. The increasing number of mobile device users has created opportunities to develop mobile apps for delivering health interventions [1]. Moreover, mobile apps can assist people with mental health issues by incorporating self-monitoring, psychoeducation, self-management, and treatment options. These apps can be especially appealing because of their anonymity, ease of access, and ease of use [2].

Consequently, researchers are increasingly using mobile apps as tools for delivering health interventions. However, a key challenge is how to design interventions that are effective and acceptable to people experiencing mental health issues. We believe that the best way to design such apps is to employ the user-centered design (UCD) approach that engages intended users and involves them in the app’s design process.

In line with the UCD process, the goal of this paper is to explore the opinions, perceptions, preferences, experiences, and ideas of people who have experienced mental health issues based on self-diagnosis so that we can design a mental health app that would be engaging and effective at improving mental health and emotional well-being.

To achieve this, we conducted 6 focus groups with 32 participants. The results revealed that participants used strategies to deal with their mental health issues: (1) doing something to distract themselves from their current negative mood, (2) using relaxation exercises and methods to relieve symptoms, (3) interacting with others to share their issues, (4) looking for an external source to solve their problems, and (5) motivating themselves by repeating motivational sentences to support themselves or by following inspirational people.

Regarding the design of mental health apps, participants identified 13 unique feature ideas and 32 unique participant-generated sketches of how their ideal mental health app would look and what it should contain. The analysis revealed a core set of features, style preferences, and characteristics considered necessary by participants for a mental health app: (1) general design characteristics; (2) personalization of the app, including (3) tracking and feedback, (4) live support, and (5) a social community; and providing (6) motivational content and (7) relaxation exercises are the most important features users want in a mental health app. In contrast, (8) games, (9) relaxation audio, (10) the Google map function, (11) personal assistance to provide suggestions, (12) goal setting, and (13) privacy were least requested.

This paper contributes to advancing state-of-the-art mental health apps by exploring the preferences, needs, and concerns of mental health app users. It also sheds light on opportunities for future work in this area by offering recommendations for designing mental health apps that meet the unique needs of this population.

### Mental Health Apps

Research on mental health and emotional well-being in human-computer interaction (HCI) is rapidly growing. There are different types of mental health and emotional well-being app interventions: (1) mental health apps to predict mental health issues, (2) mental health apps to improve the user’s awareness of their mental health symptoms, (3) mental health apps designed based on cognitive behavioral therapy (CBT) or meditations to relieve symptoms, and (4) mental health apps designed based on a game to reduce issues.

Some mental health mobile apps were designed to predict the affective health state of users by collecting mobility and contextual information [3]. For example, Canzian and Musolesi [4] designed a mobile app that collects mobility patterns of the users from GPS data to trace and assess 28 users’ depressive moods. They were able to identify a significant correlation between the changes in mobility metrics that were extracted from the mobility traces and the variations in users’ depressive mood. Similarly, Boukhechba et al [5] developed a mobile app to passively collect the GPS location and communication data (text messages and calls) from 54 college students over 2 weeks. They examined the correlation between the social anxiety level of students and passively collected data (GPS, text messages, and calls). They found that by using both mobility and communication patterns, they were able to predict the level of social anxiety of the students with an accuracy of up to 85%.

On the other hand, some mental health apps collect the personal data of users manually or passively to improve their awareness and understanding of their mental health issues [3]. Some studies have shown that self-tracking helps users to understand their mental health symptoms and be involved in their mental health management by improving their awareness. Consequently, this type of mental health app facilitates self-tracking by helping users keep track of their mental wellness data; the apps use these data to improve users’ awareness. For instance, Bardram et al [6] designed the MONARCA app, which is a personal monitoring app that allows users with bipolar disorder to monitor their mood and other factors. They compared the MONARCA app with paper-based forms and found that the app was easy to use and useful and increased adherence compared with paper-based forms.

There are also some mobile apps designed based on CBT. Bakker et al [7] conducted a study to evaluate a mobile app called MoodMission, which was designed based on CBT strategies for mood and anxiety issues. They found that the app improved mental well-being, the ability to cope, and self‐efficacy for people experiencing moderate depression or anxiety.

Moreover, mindfulness apps are especially popular. Laurie et al [8] conducted a study using a previously developed app called HeadSpace to understand how users use and experience mobile-based mindfulness interventions. They found that there are some barriers to using the app, including busy lifestyles, a lack of routine, strong negative emotions, and negative perceptions of mindfulness. Therefore, they concluded that developers should design mobile well-being interventions by considering people’s beliefs, affective states, and lifestyles and should make them adaptable to fit the needs of different users.

In addition, Franklin et al [9] designed a game app called Therapeutic Evaluative Conditioning (TEC) as a tool to increase aversion to self-injurious thoughts and behaviors (SITBs). The game became more challenging as the trial progressed. Users earned points as rewards for faster and more accurate performance. They conducted 3 separate studies for people with a severe history of SITB who were randomly assigned to use the mobile treatment TEC app or a control app for one month. They measured the effectiveness of the TEC app on “the frequency” of self-cutting, nonsuicidal self-injury more generally, suicide ideation, suicide plans, and suicidal behaviors. Across all 3 studies, self-cutting episodes, suicide plans, and suicidal behaviors were consistently reduced but suicide ideation was not.

### The UCD Approach

Involving users in the design process is essential to understand and incorporate their needs and preferences into the design. Some studies included intended users in the design processes to be able to design an application that was acceptable and more engaging to users. End users played a consultative role in the area of mental health and well-being intervention designs, such as improving psychological well-being [10-12], screening potential depression and supporting treatment choices [13], a web-based treatment program [14], self-management conditions [15], and a web-based mental health clinic [16].

Peter et al [11] conducted a participatory study to explore workers' perceptions, preferences, and ideas to design a mental health app that would be engaging and effective at improving emotional well-being for workers in male-dominated workplaces. They found that participants considered the available languages, ease of use, visual appeal, and offline mood as important features for a mental health app. Another study found that privacy, feedback, convenience, ease of use, personalization, and control over the amount of information were considered essential features in mental health mobile technologies used by adults [12].

In addition, Kenny et al [10] conducted 5 focus groups to explore adolescents’ needs and concerns regarding mental health apps. The results show that participants identified 8 important factors: *safety, engagement, functionality, social interaction, awareness, accessibility, gender, and young people in control*. Similarly, Todd et al [15] conducted 5 focus groups to inform the development of a web-based self-management intervention. Participants highlighted the importance of social activity, exercise, and support with self-management and noted that it would be useful to have advice tailored to their mental health state. Although much work has been done toward understanding users’ perceptions regarding the design of mental health apps, this study has mostly focused on the understanding of strategies used by participants who have experienced mental health issues for dealing with mental health issues in their lives, understanding participants’ perspectives and opinions in relation to selected mental health apps, and understanding how these strategies and ideas could be leveraged in designing a mental health app, all of which have received little attention in previous studies.

## Methods

### Study Design

We conducted a focus group study with people who have experienced mental health issues based on self-diagnosis to (1) explore the ways people manage symptoms and overcome their issues; (2) understand their opinions, preferences, ideas, experiences, and needs in relation to 2 selected mental health apps; and (3) engage them in a co-design session.

### Participants

We recruited participants by email (in both academic and nonacademic environments) and social networks (Facebook and Twitter). We conducted 6 focus groups, with a total of 32 participants (age range of most of our participants [88%] was 18-34 years; 16 males and 16 females) who had experienced mental health issues based on self-diagnosis. Each group had 4 to 7 participants and lasted 60 to 75 min. A total of 20 participants had used general health apps, whereas only 5 participants had used a mental health app to manage their mental health issues. We had a relatively diverse population in terms of gender, age, education level, and the type of mental health issues (Table 1).

**Table 1:** Demographics of 32 participants.

Demographics		Values, n (%)
**Gender**		
	Female	16 (50)
	Male	16 (50)
**Age (years)**		
	18-24	18 (56)
	25-34	10 (31)
	35-44	2 (6)
	45-54	1 (3)
	≥55	1 (3)
**Level of education**		
	High school or equivalent	12 (38)
	College diploma	4 (13)
	Bachelor’s degree	9 (28)
	Master’s degree	6 (19)
	Doctoral degree	1 (3)
**Mental health concern**		
	Stress	22 (69)
	Anxiety	7 (22)
	Depression	5 (16)
	Low mood	3 (9)
	Panic attack	2 (6)
	Worry	1 (3)
	Fear	1 (3)

### Producer

We conducted single-sex focus groups as people may not feel comfortable talking about personal issues, such as mental health, in mixed-gender groups. The moderator began the focus groups by asking questions to guide the group through topics related to the research topic while also taking a flexible approach and following unanticipated ideas that emerged during the discussion. The goal of the focus groups was to explore people’s mental health issues, needs, and concerns about mental health mobile apps, including discussing users’ perspectives on mental health mobile apps using 2 sample apps. The focus group session format was designed to unfold over 3 phases:

- Phase 1: exploring the type of mental health issues that participants have experienced and how they have managed symptoms or overcome the issue
- Phase 2: understanding participants’ perspectives, preferences, opinions, concerns, and needs in relation to 2 selected mental health apps
- Phase 3: engaging participants in a co-design workshop

The main goal of this structure was to use the first and second phase discussions to establish common ground by helping participants reflect and discuss different ways of managing their mental health issues and understanding their perceptions. These discussions were then used to spur a co-design session where each participant designed an app to help them overcome or control their mental health issues.

#### Phase 1: Exploring Users’ Experiences With Mental Health Issues

In this phase, we focused on exploring the type of mental health issues that users had experienced and how they dealt with these issues. We asked participants about their experiences with mental health issues and what they usually do to control or manage these issues. We also asked them if they had used a mobile app to help with their mental health issues.

#### Phase 2: Understanding Participants’ Perspectives, Opinions, Preferences, Concerns, and Needs in Relation to 2 Selected Mental Health Apps

We asked the participants to download and use two mental health apps—Happify and Self-Help Anxiety Management—2 days before the focus group session, giving them time to explore and have a sense of how the apps worked by using them before the focus group session. In addition, we gave a demo of the two apps in the focus group before our discussion of the apps. We chose these two apps for the following reasons: the Happify app is complex and comprehensive with a high number of behavior change strategies implemented, whereas the Self-Help Anxiety Management app is simple and employs fewer persuasive strategies based on previous work [17], and both apps were reviewed by mental health professionals and were published on the Anxiety and Depression Association of America’s website [18]. In this phase, we demonstrated the Self-Help Anxiety Management app first because it was the simpler app and asked participants about their initial reaction to the app, the things they liked most about the app, what things they liked least about the app, and the things they did not like in the app. Following this, we demonstrated the second app (Happify), followed by the same questions asked about the first app. Finally, we asked them whether they felt that gender impacted the use of mental health apps.

#### Phase 3: Engaging Participants in a Co-Design Workshop

In this phase, we provided participants with paper and a pencil. We then asked them to pretend that they were the designers of mental health apps and invited them to sketch their ideas of their ideal mental health app in words or pictures. We asked them to think about what they would like and want to see in their own mental health application. After they finished, we asked each participant to share their design and, as a group, we dialogued about the user-generated designs, discussing what participants liked and disliked in each design.

### Data Collection and Analysis

During each focus group session, we gathered data by audio recording the session with participants’ consent and through design artifacts (sketches) to better understand (1) participants’ personal ways of caring for their mental health issues, including general depression, stress, low mood, and anxiety; (2) their perceptions, opinions, needs, concerns, and ideas in relation to the selected mental health apps; and (3) how they reflected their needs, ideas, and perceptions in their own designs (sketches).

All group sessions were audio recorded and transcribed for coding. We conducted a thematic analysis to analyze our data [19]. Thematic analysis was chosen because it allowed us to analyze a large data set in a systematic manner that uncovered patterns in the text while considering the context of what participants said to more accurately inform our interpretation of the data. We followed the six-phase framework by Braun and Clarke [19] for conducting a thematic analysis: (1) becoming familiar with the data, (2) generating initial codes, (3) searching for themes, (4) defining themes, (5) iteratively reviewing themes, and (6) writing up the results.

**Figure 1:**
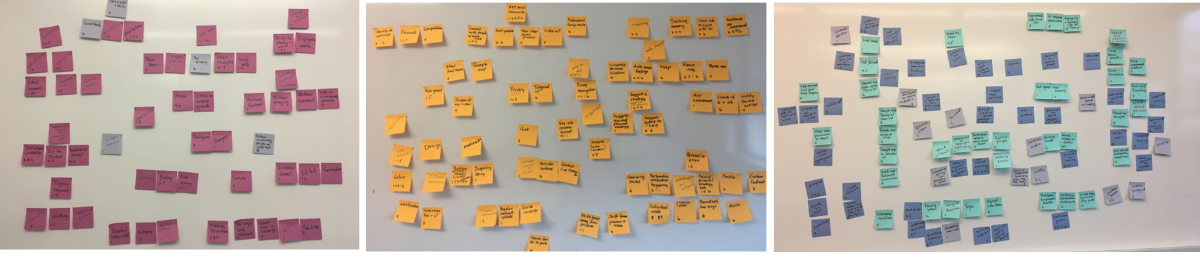
Codes generated from each phase (1, 2, and 3) in order from left to right.

**Figure 2:**
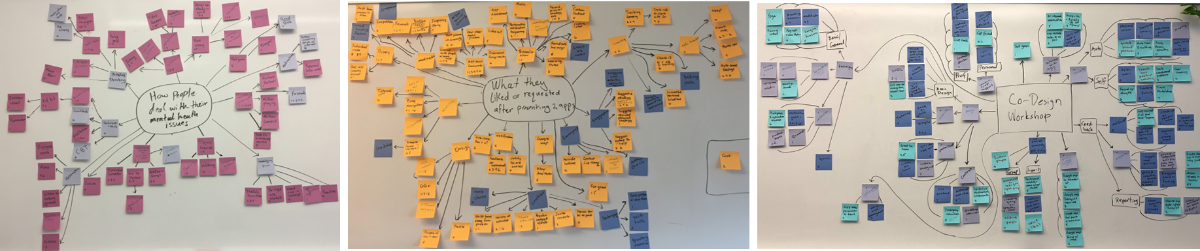
Themes generated from each phase (1, 2, and 3) in order from left to right.

Specifically, 2 researchers individually read and re-read all transcripts (iteratively) to identify codes using open coding. Following this, the 2 researchers met in a series of meetings where the codes were expanded, developed, and modified and new codes emerged. For each phase of the study group session format, we wrote each code on a separate sticky note and posted all generated codes on a large whiteboard to help identify the themes. On each sticky note, we wrote the number of times and in which focus group the theme was mentioned (Figure 1). After many iterative reviews, where we identified which themes could be revised or combined, the researchers identified a clear theme (Figure 2). Next, to further refine the themes, we presented and discussed the themes with a group of 12 researchers in the HCI Laboratory who had good knowledge of the research area. On the basis of their feedback and discussions, we generated a final iteration of the themes (the final themes are given in Multimedia Appendix 1). We presented the results of each phase in the sessions and provided quotes as specific examples from each theme within the results. Table 2 shows the frequency of occurrence of each theme that emerged in each phase. We identify participants by number (eg, P1, P2, etc) and which group each participant was associated with (eg, G1, G2, etc).

**Table 2:** Coding frame detailing the frequency of occurrence of the themes within the data.

Themes		Frequency, n (%)
**Phase 1**		
	Doing something to distract themselves	22 (22)
	Relaxation exercises and approaches	20 (20)
	Social interaction	14 (14)
	Looking for an external source of support to solve the problem	11 (11)
	Doing something to motivate themselves	10 (10)
	Helpful thinking	7 (7)
	Doing physical exercise	7 (7)
	Managing their time	6 (6)
	Religious practices	3 (3)
	Isolating themselves	1 (1)
**Phase 2**		
	The need to enhance the app design	71 (43)
	Tracking of personal data	18 (11)
	In-app social community	16 (10)
	Motivational content	14 (9)
	Personalization	11 (7)
	Providing feedback from the app	10 (6)
	The need for enhanced privacy and security features	8 (5)
	The need for relaxation approaches	7 (4)
	Providing professional support	4 (2)
	Including games for entertainment	4 (2)
	Including the Google map function	2 (1)
	Participants’ perceptions regarding gender differences when using mental health apps	32 (19)
**Phase 3**		
	General design characteristics	27 (16)
	Self-tracking of personal data	20 (10)
	Providing feedback for the users	20 (10)
	Providing live support	18 (10)
	Personalizing the app’s style and functions	18 (10)
	In-app social community	17 (10)
	Motivational content	14 (8)
	Providing relaxation exercises	10 (6)
	Including a Google map function in the app	7 (4)
	Including simple games for entertainment	7 (4)
	Providing relaxation audio	6 (3)
	Personal assistance in the app to help users	5 (3)
	Goal setting	3 (2)
	Privacy	2 (1)

## Results

### Users’ Experiences With Mental Health Issues

All participants (32/32, 100%) reported experiencing stress, anxiety, depression, panic attacks, and/or low moods at some point in their lives. They reported a variety of factors affecting their mental health. Stress and anxiety caused by *studying at a university* were the most common issues discussed and were reported by half of the participants 53% (17/32). Moreover, *being a mother* (both during pregnancy and in raising children), *money issues, losing a job, losing friends, childhood abuse, family or community issues,* and *having a large number of commitments* were mentioned as factors that contributed to them experiencing mental health issues.

Participants shared a variety of ways they had used and were using to deal with and manage their mental health issues. We identified 10 core themes related to the approaches used by the participants: *doing something to distract themselves*, *using relaxation exercises and methods*, *social interactions*, *looking for an external source of support (ie, someone or information) to solve the problem*, *doing something to motivate themselves*, *helpful thinking*, *physical exercises*, *managing their time*, *religious practice*, and *isolating themselves*.

Detailed themes are presented in Multimedia Appendix 1, and sample responses from participants in support of each theme are discussed in detail later.

### Doing Something to Distract Themselves

The participants used many strategies to distract themselves from unpleasant feelings. For instance, participants who were usually stressed, anxious, or depressed wanted to *run away* and desperately sought a way out. As a result, they spent time shopping, on social media, sleeping, focusing on work or study, or changing their environment, for instance, by physically going to a new location. All these they did as coping strategies, as illustrated in the sample comments below (comments from participants are included verbatim throughout the paper):

Every time I feel down or something, I would go to school just to run away and do something like this.

G1P1

I sleep when I feel down or stress.

G5P3

If I feel stress, I will go out to change the environment.

G3P5

In addition, when participants experienced mental health issues, they tried to entertain themselves by playing video games or mobile app games to distract themselves from their current situation, as demonstrated in some sample comments below:

I spend a lot of time on video games.

G2P1

I used to play a game when I am stressed to distract myself.

G5P2

Some people mentioned that eating to deal with their emotions was a strategy that helped them to forget their stress:

If I am depressed...I eat something that I like. I do anything that makes me feel better.

G5P2

When I am stressed, I eat a lot even if I feel I am full.

G2P1

### Relaxation Exercises and Approaches

Participants mentioned that doing relaxation practices, such as breathing or meditation, via a mobile app, YouTube, or Fitbit assisted them with reducing stress, relieving symptoms of depression, and boosting feelings of joy and well-being:

Trying breathing exercise to mitigate the stress.

G1P2

I start to use some application that has some meditation and it works.

G6P6

Furthermore, doing CBT over a long period helped participants handle panic attacks:

I did CBT for long time...so when attack comes, I know how to handle it.

G6P3

Moreover, they mentioned that practicing gratitude before bedtime for about a month assisted them in being more positive and reducing negative thoughts:

Before I sleep, I write three things that I am grateful for. For example, I am grateful for having a family...and this change me too much. I became more positive I can control my stress, fear.

G6P4

With respect to relaxation exercises employed by our participants, they highlighted that listening to natural sounds and music helped them to reduce their stress:

I have tried one app that has natural sounds such as rain which help to sleep better.

G1P2

I usually listen to music. It helps a lot.

G6P5

They also reported that strolling or sitting outside in a natural environment were other ways in which they could reduce their stress and anxiety:

If I feel depressed or sad...sometimes I like to walk next to the waterfront or in a garden.

G3P5

Moreover, making herbal tea to reduce worry and stress was also mentioned by a participant:

Sometimes I made herbal tea to relax and overcome the stress.

G1P3

### Social Interaction

Participants mentioned that talking with their mother, hearing their mother’s voice, and knowing their mother was praying for them were some of the most relieving things for them when they experienced mental health issues:

I just call my mom and I want to hear her voice and chatting, and she prays for me that is the most relieving thing.

G1P2

Because maintaining my mental health leads to my being appreciated by the people I know.

G1P12

Furthermore, spending time with friends and nurturing their partners were also mentioned as ways of reducing depression, stress, and anxiety:

I call friend and talk about it.

G1P1

In order to have a healthy life with your partner, you need to take time with him.

G1P4

Moreover, having a person with whom the participant was comfortable and could talk about a troubling issue brought the participant some relief:

Talk to a comfortable person, that helps me so much.

G5P4

### Looking for an External Source of Support to Solve the Problem

Looking for information or people that could help solve their mental health issues and help them feel relieved was another approach used by participants when they felt depressed, stressed, or anxious. Some participants joined a wellness group to share their issues and find someone who could provide them with suggestions or solutions for their issues:

I attend one session in the health and wellness center, and they teach us how to manage our stress and fear and how change our mood.

G6P4

Now I join a wellness group.

G3P3

Some participants went to see a doctor, psychologist, or counselor to find a solution for their depression or sleeping difficulties. Some of them used medication to help with their symptoms:

I went to my doctor and he said to me we need to talk we need to do something about this and I started some medications which worked for me.

G3P3

In addition, people searched for a solution to their mental health issues using Google. However, they stated that this approach was not always an accurate source of health information:

The hypochondriac is the most stuff, so I am using Google.

G6P3

### Doing Something to Motivate Themselves

In this section, participants mentioned the internal and external motivations they accessed when they felt down, stressed, or in the midst of a panic attack. One of the approaches they used was self-talk, which is an internal form of motivation people practice by repeating motivational sentences or affirmations to support them in stopping negative thoughts:

Regarding to panic attack, I just try to tell myself this is not real to get myself feel good again.

G6P7

Participants reported following inspirational people on the internet and rewarding themselves after hard work as examples of external motivations they used to encourage themselves to be more positive and reduce their mental health symptoms:

I also follow some inspirational people in the internet to motivate me.

G6P4

### Helpful Thinking

Some participants tended to think of the *bigger picture* surrounding their issues to see the benefit of the issue; this strategy helped them to reduce their stress.

I think about the overall picture. I am talking about my case for overcoming my stress...helps me so much.

G4P1

In addition, some overcame their fear by facing the things that generated that feeling:

If I am afraid from something, I do it. I found it very effective just facing your fear.

G4P2

Another strategy to avoid feeling depressed was to force themselves to do the thing that generated the depressed mood:

So, when I do poorly in school I can go to the depressive episode where I stopped going to class, I just don't feel good. The ways that I have had to deal with it...and force myself to wake up and go to the class.

G2P2

### Doing Physical Exercise

Participants stated that being physically active improved their mental well-being by reducing anxiety, depression, stress, and negative mood. Hence, they overcame their depressive feelings and anxiety by going to the gym, walking, or dancing. Some played physical games, such as soccer and billiards, which helped to release their negative feelings:

I do something I like, such as playing billiard, and soccer.

G3P2

Dancing at home makes me feel better.

G1P3

### Managing Their Time

Some participants highlighted that identifying priorities and being more rigid with their schedule helped them reduce stress and depression:

I just organize my time at least what I should do for each day to reduce my stress.

G5P5

I try to be more rigid with my schedule.

G2P2

They also mentioned that creating time to do something enjoyable and removing anything that contributed to their stress helped them in dealing with their mental health issues:

Stress is only created when you keep things inside you...so I started creating my own time I do what I want to do. I need my me time.

G1P4

### Religious Practice

Some people stated that reading religious books and praying reduced their symptoms of depression and anxiety and enabled them to cope with stress better:

I seek something religious. Do the things I believe in, reading the religious book.

G3P4

### Isolating Themselves

One person stated that when he experienced mental health issues such as feeling depressed, he preferred to withdraw and isolate himself:

I preferred to be isolated.

G5P4

### Understanding Participants’ Perspectives, Opinions, and Preferences in Relation to 2 Selected Mental Health Apps

We identified several themes related to participants’ perceptions, needs, and preferences in relation to selected mental health apps. We discuss each of these themes along with the related subthemes that follow.

#### The Need to Enhance the App Design

##### Usability

Usability is an essential factor in users’ experience of mental health apps.

- Easy to use: most participants highlighted simplicity and ease of use as important in the mental health apps that they really like to use regularly:

The home page is a very complex design. It would be better to design like icons to be easy to use.

G5P2

- Easy to navigate: participants liked to be able to find relevant or required information or app components more directly and quickly. “*I feel there are lots of things going on that it takes many steps to go there*” (G2P5).
- Organized and simple home screen: participants liked apps that were organized and had fewer details on the home screen. They preferred that app information be presented in a nonoverwhelming way. “*I liked the layout organize of the app*” (G1P3).
- Tutorial: participants expressed a preference for apps with simple instructions on how to use the app. “*If the app has a little tiny pop-up for each activates to give you brief overview, how it works so it would not be confusing*” (G2P3).

##### App Content

Participants liked having a variety of activities in the app and felt that this activated their curiosity. They liked apps that have regular content updates, and the information content in the app had to be from trusted sources. Moreover, some participants liked content that shifted their attention away from their current issues:

The information they provide looks more trustworthy than the other. There are some links I can check them out.

G1P3

This app does not have anything that let you remember your stress or depression just play games and read an article which is good.

G5P5

The app should not show stress or depression as the illness it should show as something normal that we all face in our life.

G4P2

##### Basic Design

Participants highlighted the importance of the app having a clear purpose:

Users need to understand the main purpose of the app easily.

G4P2

They also mentioned that the app name should be both simple and not include or mention or be related to any mental health issue because they do not want others to know that they are using a mental health app:

I like that it is called happify. You are clicking to happify not clicking to anxiety. Even the color is bright and happy.

G3P5

Participants preferred the color scheme of the app to be bright and calm. They also liked apps that support different languages:

It need to include other languages not only English.

G5P3

I like the app color. It is calm.

G2P2

#### Tracking of Personal Data

Participants reported 2 types of tracking: *self-tracking* and *auto-tracking*. For self-tracking, participants wanted to make their own notes and track their successful personal solutions. The app should be able to support that:

I like taking note so I can know if there is improvement or not.

G3P1

I really like the anxiety tracker which shows your progress and kind it gives yourself hope that you will get better and you can report in the things that make you up and dawn.

G2P1

For auto-tracking, participants wanted their emotions to be tracked based on the auto-tracking data of their sleep, heart rate, and phone use:

What if you have something that can track your sleeping time and maybe heart rate and know your feeling based on that.

G2P1

I do not think users can rate his/her level of anxiety. I prefer sensors to give the rate of my stress like hear-rate.

G1P2

#### Social Community in the App

Participants highlighted their desire to have a social community within the mental health app to interact socially with other users who have the same issues. This would enable them to share issues, give each other advice, and relate to other people’s experiences. They also liked anonymous communication in the social community and the ability to post a picture. Including a video calling option in the social community and having a professional monitor in the group was also suggested:

I like the community in the app, so you have someone to share with.

G3P5

Social community will be the main thing I will go for it.

G4P1

I like you can post the picture to the group.

G5P3

#### Motivational Content

Participants mentioned 2 types of motivation: motivation to encourage participants to be positive and motivation to encourage participants to use the app. Participants emphasized that the app should provide them with inspiring stories and positive news and quotes, pictures, and articles that improve their mental well-being and motivate them to be more positive:

I like the positive news. It is good to have it in the apps.

G5P2

If there is an inspiration quote, it will be good.

G6P1

Some reported that the app should allow them to gain rewards after each task they have done and compare their points with other users in the app, which would motivate them to keep using the app:

I like the points and specially if I can make it competition with other users so I can compare my points with my friends which let me practice more.

G5P1

#### Personalization

The ability to personalize the interface, for example, by customizing reminders, background, color, and music, was attractive to participants. Some also wanted to be able to add personal strategies in their profile, so the app could make personalized suggestions when they experience a crisis. In addition, some participants would like the app to administer an assessment before using the app to personalize the content based on users’ answers. However, this assessment must be short. They would also like the app to greet them personally, by their name:

I like the level and the assessment in the beginning, it can be used to make the app personalized.

G1P2

Adding personalization in your app like Hi (name), how you are feeling today?

G4P5

#### Providing Feedback From the App

Feedback from the app was divided into 2 types: notifications and suggestions.

- Notifications: participants highlighted that the app should send them periodic notifications to check on them, either randomly or at user-defined times:

There is no notification or reminder to remind me to use the app so I will forget it.

G3P3

They also highlighted the need for the app to notify them about their feelings based on their tracked personal data:

The app could do more work in the background, send a notification and check during the day how’s your day today.

G2P2

- Suggestions: participants wanted the app to provide helpful suggestions based on auto-sensed data related to their current circumstances. These suggestions could be positive articles, health advice, strategies to overcome the mental health issue, personal strategies that were previously recorded to make users feel better, or advice for the user to contact a doctor:

...for example, when you set up your account with the app, they will ask questions, what makes you happy? What is your hope? So, a month later if I feel down, they will suggest that I make a herbal tea for example.

G1P1

There should be a suggestion to contact a doctor.

G3P1

#### The Need for Enhanced Privacy and Security Features

Participants expressed a preference for the option for an individual mode, as opposed to a web-based community mode in which they can make personal notes and express their feelings within the app privately:

I will use the private mode first then after a while when I trust the app, I can change it to be community mode.

G5P4

Asking permission to access my photo, contact is good.

G3P4

They also preferred the option to use an app without creating an account:

Creating an account is something annoying for me. I do not like to create an account.

G3P5

Participants also emphasized the importance of the lock feature in mental health apps to protect their collected data and information:

There is no locking feature in the app and that increased my concern regarding privacy.

G1P3

#### The Need for Relaxation Approaches

Participants reported the importance of practicing relaxation approaches to reduce unpleasant feelings and feel better. Self-talking and breathing exercises were the strategies that users wanted to have in their mental health apps:

I like the breathing exercises in the app.

G1P2

The app should allow users to speak to themselves.

G6P4

#### Providing Professional Support

Participants suggested that the app should provide access to mental health professionals’ support by either providing the contact details of doctors, coaches, and a suicide crisis hotline or by providing live therapists who can be accessed to respond to their concerns:

If there is icon that you can contact doctor, clinic, it will be good.

G3P2

There should be a hotline for suicide.

G2P3

#### Including Games for Entertainment

Participants reported that apps need to include fun games as a way to reduce stress:

The app should have entertainment such as game not only record your notes and mood.

G5P5

I like the game idea it is a kind of entertainment.

G1P3

#### Including Google Map Functionality in the App

It was suggested that mental health apps should have a Google map functionality that shows centers and communities for mental health support that are geographically near the user. Therefore, when users feel down, stressed, or depressed and use the app, they can easily find a physical community for support:

If there is icon that show the center or society that help people for example the app has Google map and show the mental health center and society that nearby users.

G5P4

#### Participants’ Perceptions Regarding Gender Differences in Using Mental Health Apps

Some participants believed that men would be less likely to use mental health apps because women tend to look for ways to express their feelings, whereas men tend to hide their feelings and pretend that nothing affects them. However, other participants felt that mobile apps could motivate and help men to express their emotions. Others felt that differences in using mental health apps are less gender related and more likely dependent on the personality type of the users:

I think women use mental health app more because they are more emotional, and the man can keep his emotion and pretend nothing affects him.

G3P3

I think all men and women use mental health app but it depends on the personality. There may be some women do not like to use an app and as well as men, so it depends.

G2P1

### Co-Design Workshop

The co-design workshop produced 32 participant design sketches and an extensive set of app design components, including user-preferred features, functionalities, and characteristics that mental health app users would like to use. The researchers identified 13 major themes from the co-design workshop. We discuss these themes below.

#### General Design Characteristics

Participants mentioned simplicity and ease of use when explaining their design. They highlighted that this would allow them to learn the app quickly:

I kept mine as simple as possible. I was thinking of a person who does not like to be on the phone or just needs the basics.

G2P2

In addition, they reported that mental health apps should include a tutorial or instructions for how to use the app.

The important thing for me is to show the users how to use the app.

G5P3

Participants emphasized that mental health apps could provide (1) credible information in a way that is not overwhelming, (2) a variety of activities, and (3) crisis information support:

Any information should be from a qualified person, not anybody can post the information.

G3P3

I would like to have a variety of activities such as yoga, breathing.

G1P3

It provides ... emergency and instruction of what users should do.

G4P4

Participants highlighted that mental health apps must have a simple name. Others suggested that the app’s name must not mention any mental health issues within it:

I suggested to name the app ‘solve your problem’ for example.

G4P3

Moreover, it was recommended that the app should show the user’s achievements, what they like, and what they dislike in their profile:

...*it has a profile of what you like and dislike and activities that make you happy and people that make you happy, things like that*. [G2P2]

Some users suggested creating an account within the app to store their information, whereas others disagreed with this idea. Participants found a point of agreement with the idea of making the account optional, and the app could provide more activities and content if the user chose to create an account and formally log in:

The app shows the analysis locally that user is using the specific relaxation activities, then it suggests for user that If you log in, you will be able to access more relaxation activities.

G4P1

Participants would like to have favorite lists, enabling them to save their favorite videos and activities on the app so they could find them quickly later:

You can like the video, article and watch them again.

G4P3

#### Tracking of Personal Data

**Figure 3:**
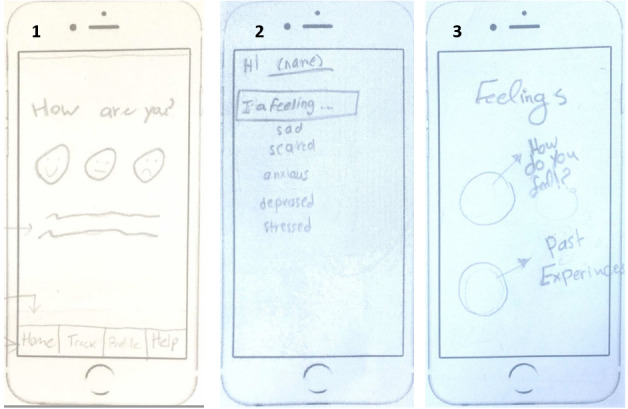
Three implementation ideas of a tracking feature were generated by participants: (1) simple tracker using a set of smiley faces, (2) using a set of emotion words, and (3) labeling one’s emotions in detail.

The tracking feature emerged in all 6 co-design sessions; 63% (20/32) of participants highlighted self-monitoring as an important feature in the app. There were various ways in which tracking was visualized by the participants in the co-design workshop. Specific ideas about how to design the tracker varied, with some participants indicating that they would like to have an auto-tracker to track their sleep time, heart rate, time spent on the phone, and in-app achievements. Moreover, some people suggested that the app could auto-track users’ emotions based on their heart rate and breathing. Others suggested that the app should provide the ability to track their mood by using a set of smiley faces or emotion words or by labeling one’s emotions in detail to identify the factors that could contribute to mental health issues (Figure 3):

The app asks users what they feel and track other problems that may lead to depression such as obesity, social problem.

G3P1

If the app can know your emotions through heart-rate and breathing, it would be great.

G4P4

The app could also track users’ alcohol consumption rates:

The app can track your drink and money to see how you are coping over time.

G4P6

Others did not like this idea of self-tracking because of a high probability of error and consistent data input and preferred the app to predict their emotions based on the auto-tracking of their heart rate:

It could be auto-tracking for blood pressure, heart-rate and knowing the emotions and providing recommendation based on users’ feelings.

G5P2

Others suggested that the app should provide the ability to take note of emotions in detail to improve self-awareness:

You can track your feeling and write what make you feel down.

G5P1

#### Providing Feedback for the Users

We divided the app feedback into 3 types: notifications, suggestions, and reporting.

##### Notifications

Participants provided a variety of ideas for notifications and wanted the app to remind them of positive things in general and good things happening in their lives as a way of redirecting their attention away from the negatives:

If you feel stress, your app reminds you of the good thing happening to you which can help you to relax.

G4P3

The last thing is reminder to tell me a nice thing, everyone wants to feel good.

G6P7

They would like the app to remind them to track their mood and activities. They would also like the app to check in on them to see if they need help, either through auto-track sensing or at random times:

I like the notifications because the apps that do not have a notification, are forgotten.

G4P2

App can check me out at random time.

G6P5

Participants would like to be notified if they had made progress toward overcoming their mental health issues by showing motivational messages. For example, “*If the app sees you have done good progress toward the anxiety, it says ‘Wow, well done!*’ [G1P2].

##### Suggestions

**Figure 4:**
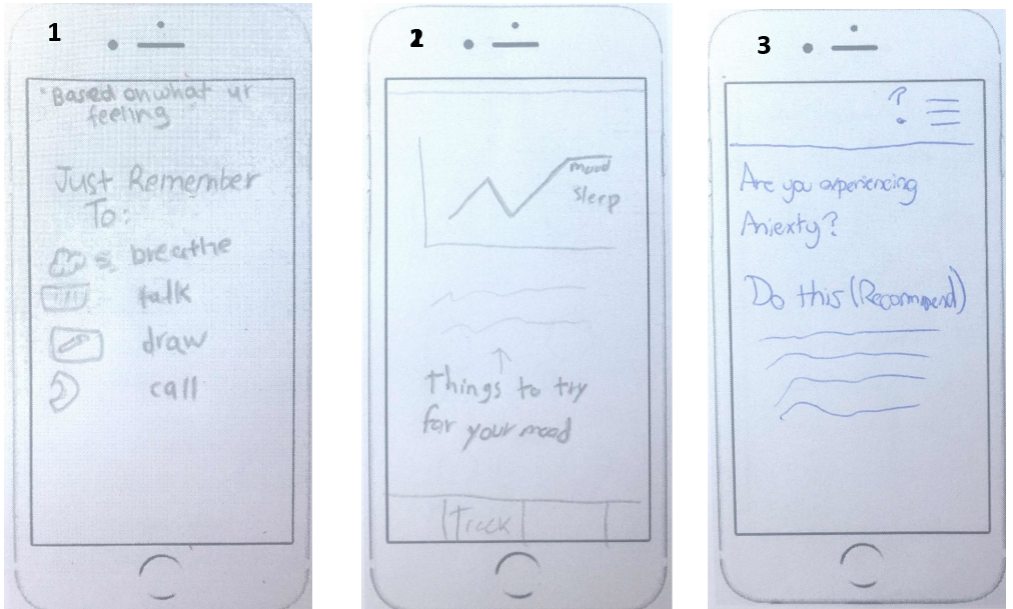
Three implementation ideas of a suggestion feature: (1) suggesting activities based on the moods entered, (2) providing suggestions to improve one’s mood based on collected data, and (3) providing general suggestions.

Participants would like the apps to provide suggestions on what to do based on their feelings. The suggestions could be built into the app, things such as reading positive articles, health advice, or news, or could be things users liked or that they entered previously into the app as things that worked for them (Figure 4):

The app can track sleep or mood and immediately diagnosis and provide you with some suggestions on what you can do to feel better based on the data the app has about you.

G2P2

##### Reporting

Participants highlighted that they would like the apps to provide a summary report of their data and/or progress that could be shared with their therapist:

The app shows a summary of the results.

G6P6

The therapist keeps up to date and see how you are progressing or your behaviors.

G2P1

Moreover, some participants expressed that they would like it if their therapist could access their data if they were given permission:

Therapist can access my data if I allow it because I try to take note for each thing, having everything in the app will make it easy to show my doctor.

G3P3

#### Providing Live Support

**Figure 5:**
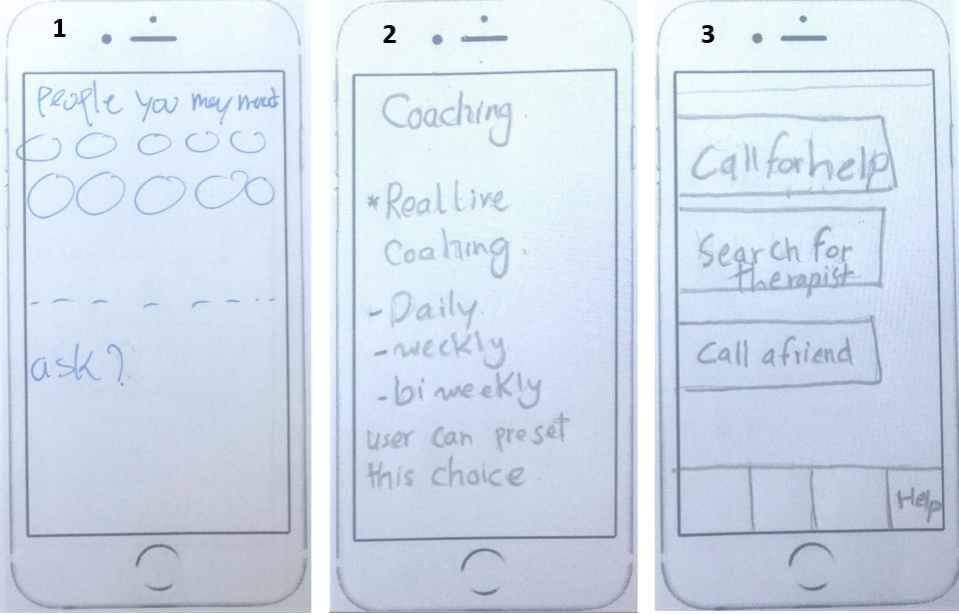
Three implementation ideas of a live support feature: (1) including therapists that users can contact and the ability to text them; (2) the ability to set up meetings with a live coach, and (3) the ability to call for help, contact a friend, or search for a therapist.

Participants highly valued live support, either personal or professional; 56% (18/32) of participants included live support in their ideal design. Figure 5 shows 3 implementation ideas of a live support feature that were generated by participants.

Personal live support allows users to provide contact details for friends and family to contact for support when necessary. It also allows for a way to contact a stranger through the app:

The app includes help information: call for help first option, search for a therapist is the second option, call a friend.

G2P2

The app allows users to call a friend or to speak to someone who use the app even a stranger that can help.

G6P7

Professional live support allows users to contact a doctor or therapist in their area. It also provides live therapists and coaches who can respond to their concerns. Participants would be willing to pay for sessions with a live therapist when necessary:

The last icon is about contact the therapist through online and it could have fees.

G5P1

#### Personalizing the App’s Style and Functions

**Figure 6:**
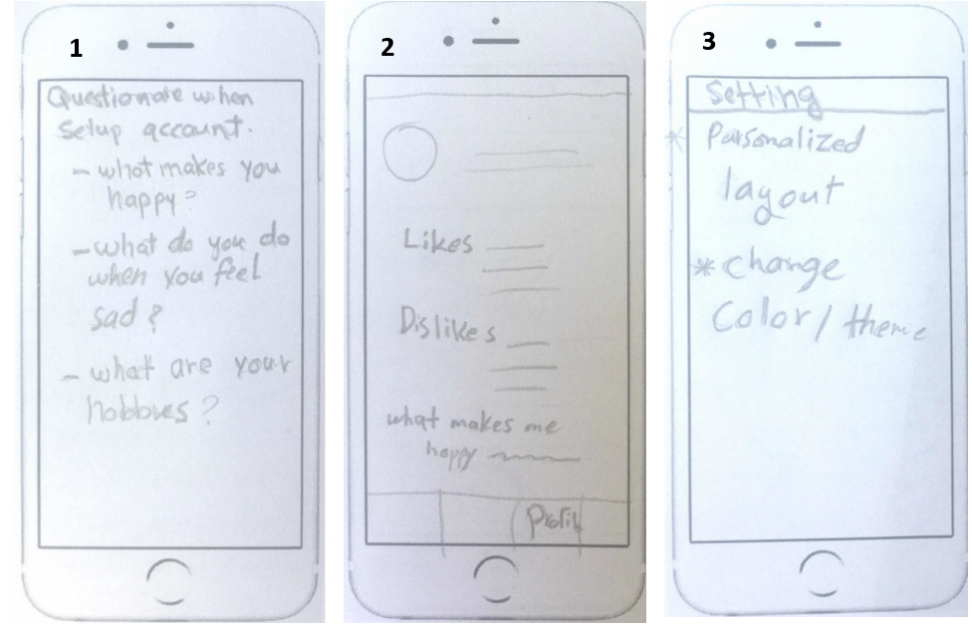
Three implementation ideas of a personalization feature: (1) asking a set of questions when setting up the app; (2) including a profile with users’ likes, dislikes, and what makes users happy; and (3) customizing the layout and theme of the app.

The ability to personalize the app’s style and function was attractive to 56% (18/32) of the participants. Figure 6 shows 3 implementation ideas of a personalization feature that were generated by participants. For example, they would like to customize reminders, background, color, and music and create their own personal in-app strategies.

Participants would like to have an initial brief questionnaire that could be used to personalize their app experience based on the user’s responses to the questions. For example, G1P1 stated, “*The app can have a brief questionnaire to get information to personalize the app*.”

Participants also highlighted the need for the app to provide them with solutions based on selected feelings or issues. For example, G4P2 stated, “*The apps provide you with buttons with different issues, you can click on which one that you need help with, and it can direct you where you can find different solutions to the issue*.”

Some participants said that it would be nice if the app could greet the user by name:

App can say hey with user’s name.

G4P4

Some participants would like the app to provide music or religious phrases based on what the users are currently feeling:

There could be different options for different religions, so I can choose based on my religion and pop-up phrase to inspire me based on my feeling.

G6P5

#### Social Community Feature

Participants reported a desire to include a social community within mental health apps so that they could interact with other users who have the same issues, share their problems, and give each other advice.

The suggested design implementations for the social community were as follows.

The social community could have different groups created based on mental health or life issues. Users are able to follow any one of these groups by searching for issues they are facing or by answering an assessment questionnaire that directs users to the right group. Moreover, users could create a group for a specific issue and add people to it:

In chatting can have different groups and different topics or I can create a group for depression in the school and anyone is interested can get in.

G2P4

The app asks you questions such as what is your issue? and based on this assessment, it takes you to the group which has the same issues, then you can share your issues and do activates together.

G5P3

The social community could have one-on-one chatting to talk to a person who has the same issue. The idea is that users can send a request and, if the request is accepted, they can chat together.

**Figure 7:**
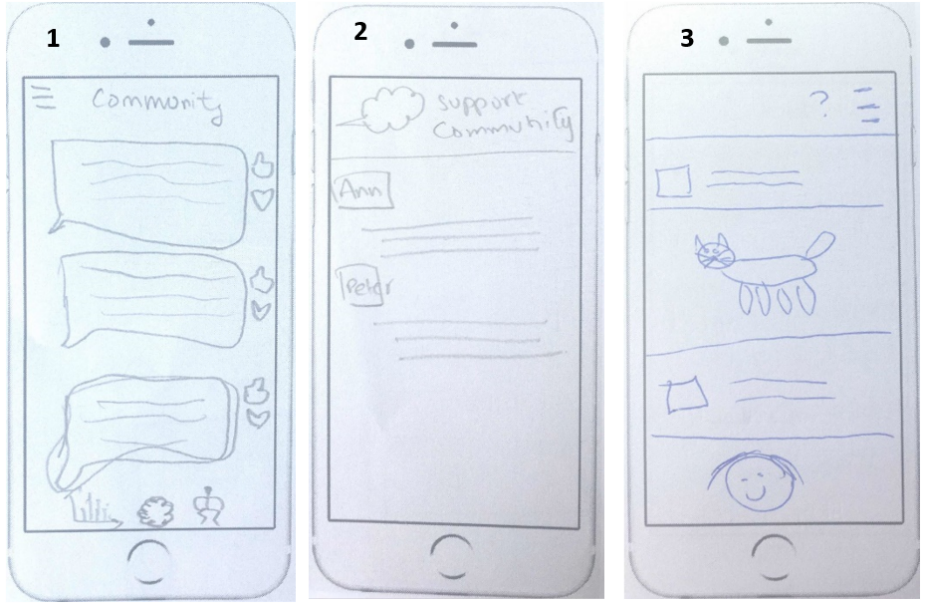
Three implementation ideas of a social support feature were generated by participants: (1) ability to reply to a comment, (2) ability to chat privately, and (3) ability to upload pictures in the chat room.

The social community could also offer voice calling; however, it should be anonymous. It could also allow users to reply to a comment and upload pictures in the chat room. Figure 7 shows 3 implementation ideas of social community features:

If user finds a person who has the same issue, he makes a match, if the person accepted, then they can do a voice call but it should be anonymous.

G5P2

Enter your issues and the app shows you the people who has the same issues and their solutions.

G4P4

#### Motivational Content

Participants expressed a desire for positive motivational quotes or statements that are updated regularly. They would also like positive news available in different formats (such as text, audio, and video) and religious inspirational quotes in the apps to make them feel better. They also reported that the app giving them rewards after any accomplishment would motivate them to keep using the app:

The introduction page has simple motivation statements such as you are the best, be confident. It should be updated every 6 hours or make it daily.

G2P4

Positive news is a good idea to have in the app which helps, and it can be in different format. Reading for people who like to read, video for people who like to watch and audio for people who like to listen.

G2P1

#### Providing Relaxation Exercises

Participants reported that the app could contain approaches for symptom relief, such as breathing exercises, yoga, recording thoughts, a forgiveness feature, and a regret feature. However, some people disagreed about the usefulness of a regret feature, as this could force them to remember unpleasant and depressive experiences and this would not make them feel better:

I want to record my thoughts. Sometimes when you have anxiety, you need someone to talk to, so recording something make you feel like you have spoken to someone.

G6P7

I would like to add forgive feature to forgive people who hurt us, and we can do it monthly.

G6P4

#### Including a Google Map Function to Find Clinics Near Users

Participants emphasized the idea of having Google map functionality that shows available therapists and communities for mental health support near the users. In addition, participants wanted a map that shows organizations in need so they could volunteer and improve their feelings by helping others:

It can have something like Google map to show the therapists that are near to me. It can pop up and users can select and contact.

G2P4

If the app tells me to help this charity or community by showing them in the Google map.

G6P3

#### Including Simple Games

Participants reported that the content of the app should be fun and interactive; thus, they included games in their design:

There are four icons in home page as main categories including an icon for a game.

G1P2

#### Providing Relaxation Audio

Participants highly valued the idea of having relaxation audio such as music, natural sounds, and worship and religious audio clips:

The first thing I want is sound or music just to help me calm down.

G6P7

For me personally, I like to hear worship sounds.

G6P5

#### Providing Personal Assistance in the App to Help Users

Some participants reported that using advanced artificial intelligence (AI) technologies in mental health apps could help to improve their mental health issues by providing individualized emotional support, assessment, and advice based on their recorded data:

You can click on and talk to your phone and tell it that something happened or that you feel bad. This could be simple or complex information and the app can understand you and give you a quick solution on what to do or suggest that you talk to your friend.

G4P6

Having a voice in the app talk to me and ask question. It is kind of robotic.

G6P5

It could be AI that responds to me and if the situation is serious it could tell me to contact a therapist.

G5P4

#### Goal-Setting Feature

Participants would like to set up their health-related goals. The app could show them how many days they have left to complete their goal, which can motivate them to finish their task and reduce their stress:

Goal setting shows how many days left to achieving the goal.

G2P3

#### Privacy Feature

Participants were highly valued when the app requested permission to access the personal data on their phone. Some would like to have a clear privacy policy that shows how their data will be protected:

There should be privacy agreement that there is no sharing of users’ information.

G1P1

## Discussion

This study aims to explore the perspectives, preferences, opinions, and needs of those who have experienced mental health issues such as stress, general depression, anxiety, panic attacks, and low mood. Given the well-documented difficulties engaging people in promoting mental health, such perceptions are likely to be pivotal to develop a successful app for mental health interventions.

Our results highlight that people who have experienced mental health issues use existing strategies for dealing with mental health issues in their lives, and these strategies should be leveraged in designing a mental health app.

Participants’ ideas were organized into 14 overarching themes showing what they want in a mental health app. Most of the desired characteristics, features, and design implementations emerged in all 6 of the group sessions.

Regarding app design, participants emphasized that essential characteristics for mental health apps are the app being simple, easy to use, and easy to navigate. These characteristics were unsurprising and consistent with those given in previous studies [20].

Moreover, allowing users to customize the app to suit their individual needs and preferences is an excellent way to improve user satisfaction, engage them, and, hence, reduce the currently high attrition rates associated with mental health apps [21,22]. Moreover, in other health domains, personalized health interventions have been found to be more effective than those employing a one-size-fits-all approach [23,24] and, in depressive and anxiety disorders, this is even more important [25,26]. Therefore, it is not surprising that the participants of this study, who have experienced mental health issues, would like to personalize mental health apps to suit their needs so that they can benefit from individualized solutions.

Participants with mental health issues usually look for someone to help them solve their issues without judging or stereotyping them. Therefore, including a social community and access to live support in the app reflects this approach to dealing with their issues in real life, as highlighted by the participants. This finding is similar to that of Alqahtani et al [20], who found that including social support and emergency contacts in a mental health app were appreciated by users. Therefore, sharing issues, advice, and one’s solutions with people who have the same issue and the ability to contact professional support, family, or even friends are important for people who have experienced mental health issues. Unsurprisingly, because of the stigma associated with mental health issues, few participants suggested that contacting and talking to a stranger who has no possibility of knowing them is preferred, which would help reduce their issues.

Although participants reported a need for tracking features in mental health apps, their ideas varied. Some participants would like their emotions to be predicted based on the app auto-tracking their vital signs, such as heart rate, blood pressure, and breathing, or based on auto-tracking their phone use as they do not trust their ability to self-track. In contrast, others suggested that the app could have self-tracking to enable users to track their positive emotions, successful personal solutions, and personal notes, all of which can improve their awareness. It is worth mentioning that self-tracking is the most frequently implemented persuasive strategy in the available mental health apps [17].

The feedback theme is a combination of 3 features: notifications, suggestions, and reports. Notifications and reminders are highlighted as very important features to remind users to use the app. Apps without notifications or reminders can be easily forgotten. However, this feature should be customizable so that users can make it random or set up the frequency of reminders; they should also be able to disable it if they do not want reminders. Receiving suggestions based on a current feeling or progress toward the user’s goal is a valuable feature to help users manage their symptoms and motivate them to keep using the app. Moreover, the ability to share a summary of the report with their health provider will save them time and help them facilitate an accurate diagnosis.

Participants stated that they often like to motivate themselves by repeating a motivational sentence, following inspirational people on the internet, or stopping negative thoughts. Therefore, including motivational content, such as positive stories, news, and inspirational quotes in mental health apps will improve users’ mental well-being. Moreover, they stated that rewarding themselves after hard work is a way to motivate themselves; therefore, including rewards in the app as a type of motivation is critical.

Playing mobile/video games is one of the strategies that some participants tend to use as a way of distracting themselves from current negative feelings and as a way of entertaining them. Therefore, including simple games in the app is important to entertain users and distract their attention from negative thoughts. In addition, including relaxation exercises and audio clips in the mental health app will bring participants some relief.

Participants have different ways of using mental health apps. Some people prefer to use them only when they are in a crisis: From their perspective, the app should have specific components that help them relax and calm down. Alternatively, others would like to use the app on a daily basis: they suggested a variety of ideas to motivate them to use the app daily, such as reminders and tracking. Textbox 1 shows the implementation ideas that emerged from the focus group study.

**Implementation ideas that emerged from the focus group study prioritize the list numbers for each category based on importance.**

- Track personal data to improve mental health
  Predicting emotions based on the auto tracking of their sleep, heart rate, breathing rate, and phone use data
  Auto tracking of users’ sleep time, heart rate, time spent on the phone, and in-app achievements
  Tracking of mood and other factors that can contribute to mental health issues
  Tracking of user’s notes and successful personal solutions
  Tracking users’ alcohol consumption rate
- Provide access to a social community group
  Having different groups created based on mental health or life issues
  The ability to create a group for specific issues and inviting people
  The ability to follow any one of these groups by searching for issues users are facing
  Ensuring and maintaining users’ anonymity while communicating in the social community
  Providing an assessment questionnaire to direct users to the right group
  Including one-on-one chatting in-app via sending a request and users chatting together in case of acceptance of the request
  The ability to post a picture in a group
  The ability to make a video/voice call
  Adding some basic rules to the community, such as respect and prohibit threats or bullying
  Adding a block option to block undesirable contacts
- Provide motivational content
  Including positive news available in different formats, such as text, audio, and video
  Including positive motivational quotes or statements that are updated regularly
  Providing religious inspirational quotes and inspirational stories
  Providing rewards after any accomplishment to motivate users to keep using the app
- Personalize the app’s styles and functions
  The ability to customize reminders, background, color, and music
  Providing a short assessment before using the app to personalize the app’s content based on users’ answers
  A personal app greeting (greeting users by name)
  Providing users with solutions based on selected feelings or issues
  Providing music and/or religious phrases based on what users are currently feeling
  The ability to add personal strategies in their profile so the app can make personalized suggestions when they experience a crisis
- Provide reminders and notifications
  Sending periodic notifications to check on users, either randomly or at user-defined times
  Reminding users to track their mood and activities
  Notifying users about their feelings based on their tracked personal data
  Reminding users of positive things in general and good things happening in their lives as a way of redirecting their attention from the negatives
  Notifying users if they have made progress toward overcoming their mental health issues by showing motivational messages
- Provide suggestions for users
  Providing suggestions on what to do, such as positive articles, health advice, strategies to overcome the mental health issue, personal strategies that were previously recorded to make users feel better, or advice for users to contact a doctor, based on their feelings or auto-sensed data related to their current circumstance
- Allow ability to generate a report
  Providing a summary report of their data and/or progress that can be shared with their therapist
- Include games to distract and entertain them
  Including a simple game (such as a puzzle or a focus on positive words) in the mental health app for distracting users’ attention from their current (negative) mood and entertaining them
  Including simulative games such as memory cards, web-based games with other participants
- Provide goal setting
  The ability to set up health-related goals and show users how many days they have to complete their goal
- Provide relaxation exercises and audio clips
  Providing relaxation exercises such as breathing, meditation, gratitude, recording thoughts, CBT, forgiveness, and yoga exercises
  Including relaxation audio such as music, natural sounds, worship, and religious audio clips
- Provide a doctor or therapist, family, and friends as contacts for external support
  Providing a suicide crisis hotline
  Allowing users to provide contact details for friends and family to contact for support when necessary
  Allowing users to contact a doctor or therapist in their area
  Providing Google Map functionality that shows available therapists and communities for mental health support near users
  Providing live therapists and coaches who can respond to their concerns
  Providing users with a way to contact a stranger through the app
- Include personal assistance in the app
  Providing individualized emotional support, assessment, and advice based on their recorded data using artificial intelligent
- Improve the privacy and security of the app
  Asking permission to access the user’s photos, contacts, and so on
  Including a clear privacy policy that shows how their data will be protected
  Offering a lock feature in the mental health app to protect users’ collected data and information
  Offering an option for an individual mode, as opposed to web-based community mode, where they can make personal notes and express their feelings within the app privately
  Providing users with the option to use the app without creating an account
- General design preferences
- Usability
- Making the app easy to use and easy to navigate
- Including instructions on how to use the app

Making the home screen organized to simplify it

Content

- Providing a variety of activities
- Updating the app’s content regularly
- Providing credible information
- Creating app content that shifts users’ attention away from their current issues

Basic design

- Making the color scheme of the app bright and calm
- Making the purpose of the app clear
- Including profile and favorite lists
- Making account creation optional or offering a choice to sign up with Facebook or Google account
- Making the app’s name simple and not including any mental health issue in the name

### Design Recommendations

On the basis of our findings, we offer concrete app design recommendations to improve users’ adherence to, engagement with, and ability to benefit from mental health and well-being apps:

1. Developers should formally evaluate their app to ensure that the app is usable. It is also critical that only credible information is presented within the app. These factors are very important for improving users’ trust and engagement and reducing attrition rates.
2. Developers should allow users to adapt some app features and functions, such as including a coping strategy that works for them; customizing reminders and notifications; and adapting the font size, font color, background, and layout to suit their preferences. Moreover, they should also be able to personalize the app based on their personal collected data via initial questions and data tracking. This will enhance the overall usability of the app and ensure a personalized experience for each user.
3. The app should provide a form of social support with anonymous communication. In addition, access to professional and personal live support should be provided in case of depression or suicidal feelings.
4. The app should provide motivational articles, news, and quotes to improve mental health and well-being. Tracking and feedback features should be provided in mental health apps. The tracking implementation should match the purpose of the app, specifically whether it should predict emotions based on auto-sensed data or by using self-tracking to improve the user’s awareness through self-reflection while encouraging users to add more details about the causes of their emotions.

### Limitations

The limitation of this study is that most participants in this study were educated people, meaning their responses may not be generalizable to less educated people.

### Future Work

Tracking users’ data was the most common feature across users’ design sketches. Nowadays, predicting and tracking mental health (eg, anxiety) is possible using wearable electroencephalogram (EEG) devices. Therefore, an interesting area for future work would be to explore the possibility of a wearable EEG that accompanies an app for discovering mental health issues that can be recognized or predicted easily and what type of matrixes can be extracted from raw data to describe mental health issues.

### Conclusions

This research is part of a project that aims to develop and evaluate the effectiveness of a mental health mobile app for promoting mental health. Findings from this study generated insight into people’s perspectives, opinions, and preferences regarding the use of mobile apps to support mental health and how such apps should be designed.

Through a 3-phase study with 32 participants, which involved the phases of exploring users’ experiences with mental health issues; understanding participants’ perspectives, opinions, and preferences in relation to 2 selected mental health apps; and a co-designing session, we identified 14 unique feature ideas and generated 32 participant design sketches of an ideal mental health app. Our findings revealed that participants used strategies to deal with their mental health issues: (1) doing something to distract themselves from their current negative mood, (2) using relaxation exercises and methods to relieve symptoms, (3) interacting with others to share their issues, (4) looking for an external source to solve their problems, and (5) motivating themselves by repeating motivational sentences to support themselves or by following inspirational people. Moreover, regarding the design of the mental health app, participants identified the following: (1) usability of the app; (2) *personalization of the app, including* (3) *tracking and feedback,* (4) *live support, and* (5) *social community; and providing (6) motivational content* and (7) relaxation exercises are the most important features users want in a mental health app. In contrast*, (8) games, (9) relaxation audio, (10) the Google map function, (11) personal assistance to provide suggestions, (12) goal setting,* and (13) *privacy preservation* were surprisingly the least requested features. Understanding end users’ needs and concerns about mental health apps will inform the future design and development of mental health apps that are usable, useful, accepted, and successfully used by the target audience to promote mental health and emotional well-being.

## Appendix

Multimedia Appendix 1 Final Themes of each Phase (1, 2, and 3).

## Abbreviations

AI: artificial intelligence
CBT: cognitive behavioral therapy
EEG: electroencephalogram
HCI: human-computer interaction
SITB: self-injurious thoughts and behavior
TEC: Therapeutic Evaluative Conditioning
UCD: user-centered design

## References

[1] Kumar S., Nilsen W.J., Abernethy A., Atienza A., Patrick K., Pavel M., Riley W.T., Shar A., Spring B., Spruijt-Metz D., Hedeker D., Honavar V., Kravitz R., Lefebvre R. C., Mohr D.C., Murphy S.A., Quinn C., Shusterman V., Swendeman D. (2013). Mobile health technology evaluation: the mHealth evidence workshop. Am J Prev Med.

[2] Klasnja P., Pratt W. (2012). Healthcare in the pocket: mapping the space of mobile-phone health interventions. J Biomed Inform.

[3] Sanches P, Janson A, Karpashevich P, Nadal C, Qu C, Daudén Roquet C, Umair M, Windlin C, Doherty G, Höök K, Sas C (2019). HCI and Affective Health: Taking stock of a decade of studies and charting future research directions.

[4] Canzian L., Musolesi M. (2015). Trajectories of depression: Unobtrusive monitoring of depressive states by means of smartphone mobility traces analysis.

[5] Boukhechba M., Huang Y., Chow P., Fua K., Teachman B. A., Barnes L. E. (2017). Monitoring social anxiety from mobility and communication patterns.

[6] Bardram J. E., Frost M., Szántó K., Faurholt-Jepsen M., Vinberg M., Kessing L. V. (2013). Designing mobile health technology for bipolar disorder: a field trial of the MONARCA system.

[7] Bakker D., Rickard N. (2019). Engagement with a cognitive behavioural therapy mobile phone app predicts changes in mental health and wellbeing: MoodMission. Aust Psychol.

[8] Laurie J, Blandford A (2016). Making time for mindfulness. Int J Med Inform.

[9] Franklin J.C., Fox K.R., Franklin C.R., Kleiman E.M., Ribeiro J.D., Jaroszewski A.C., Hooley J.M., Nock M.K. (2016). A brief mobile app reduces nonsuicidal and suicidal self-injury: Evidence from three randomized controlled trials. J Consult Clin Psychol.

[10] Kenny R., Dooley B., Fitzgerald A. (2016). Developing mental health mobile apps: Exploring adolescents' perspectives. Health Informatics J.

[11] Peters D., Deady M., Glozier N., Harvey S., Calvo R.A. (2018). Worker Preferences for a Mental Health App Within Male-Dominated Industries: Participatory Study. JMIR Ment Health.

[12] Proudfoot J., Parker G., Hadzi Pavlovic D., Manicavasagar V., Adler E., Whitton A. (2010). Community attitudes to the appropriation of mobile phones for monitoring and managing depression, anxiety, and stress. J Med Internet Res.

[13] Gordon M., Henderson R., Holmes J.H., Wolters M.K., Bennett I.M., SPIRIT (Stress in Pregnancy: Improving Results with Interactive Technology) Group (2016). Participatory design of ehealth solutions for women from vulnerable populations with perinatal depression. J Am Med Inform Assoc.

[14] Monshat K, Vella-Brodrick D, Burns J, Herrman H (2012). Mental health promotion in the Internet age: a consultation with Australian young people to inform the design of an online mindfulness training programme. Health Promot Int.

[15] Todd N.J., Jones S.H., Lobban F.A. (2012). "Recovery" in bipolar disorder: how can service users be supported through a self-management intervention? A qualitative focus group study. J Ment Health.

[16] Ospina-Pinillos L, Davenport TA, Ricci CS, Milton AC, Scott EM, Hickie IB (2018). Developing a Mental Health eClinic to Improve Access to and Quality of Mental Health Care for Young People: Using Participatory Design as Research Methodologies. J Med Internet Res.

[17] Alqahtani F., Al Khalifah G., Oyebode O., Orji R. (2019). Apps for Mental Health: An Evaluation of Behavior Change Strategies and Recommendations for Future Development. Front. Artif. Intell.

[18] Mental Health Apps. Anxiety and Depression Association of America.

[19] Braun V., Clarke V. (2006). Using thematic analysis in psychology. Qualitative Research in Psychology.

[20] Alqahtani F., Orji R. (2020). Insights from user reviews to improve mental health apps. Health Informatics J.

[21] Arean P.A., Hallgren K.A., Jordan J.T., Gazzaley A., Atkins D.C., Heagerty P.J., Anguera J.A. (2016). The Use and Effectiveness of Mobile Apps for Depression: Results From a Fully Remote Clinical Trial. J Med Internet Res.

[22] Roepke A.M., Jaffee S.R., Riffle O. M., McGonigal J., Broome R., Maxwell B. (2015). Randomized Controlled Trial of SuperBetter, a Smartphone-Based/Internet-Based Self-Help Tool to Reduce Depressive Symptoms. Games Health J.

[23] Orji R., Mandryk R.l., Vassileva J. (2017). Improving the Efficacy of Games for Change Using Personalization Models. ACM Trans. Comput.-Hum. Interact.

[24] Orji R (2014). Design for behaviour change: a model-driven approach for tailoring persuasive technologies.

[25] Carlbring P., Maurin L., Törngren C., Linna E., Eriksson T., Sparthan E., Strååt M., Marquez von Hage C., Bergman-Nordgren L., Andersson G. (2011). Individually-tailored, Internet-based treatment for anxiety disorders: A randomized controlled trial. Behav Res Ther.

[26] Silfvernagel K., Carlbring P., Kabo J., Edström S., Eriksson J., Månson L., Andersson G. (2012). Individually tailored internet-based treatment for young adults and adults with panic attacks: randomized controlled trial. J Med Internet Res.

