# Supplementary figures and images for: Co-Designing a Mobile App to Improve Mental Health and Well-Being: Focus Group Study
Source: JMIR Form Res. 2021 Feb 26;5(2):e18172. doi: 10.2196/18172 (PMC7954656; doi:10.2196/18172)

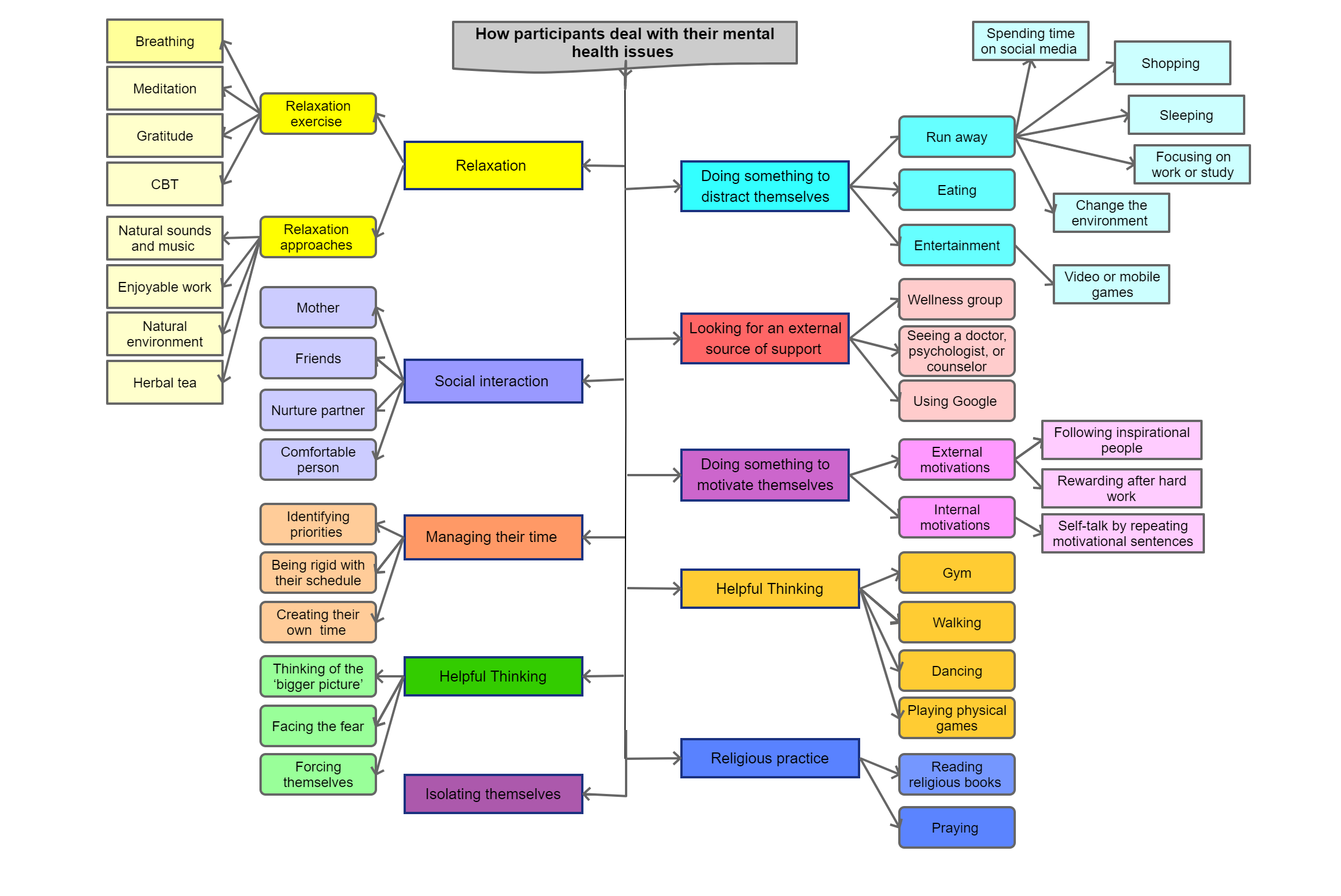


Appendix: Final Themes of Phase 1


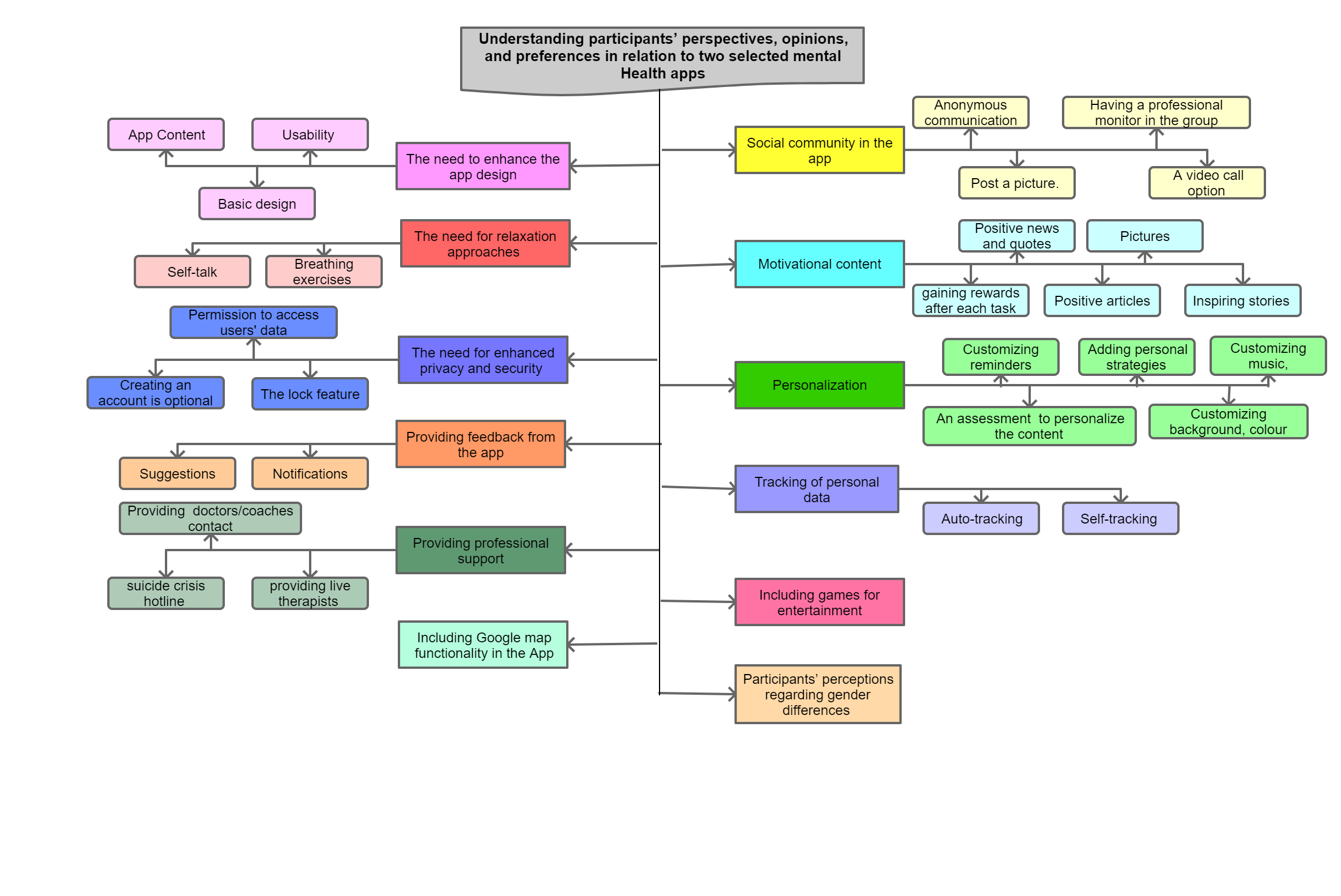


Appendix: Final Themes of Phase 2


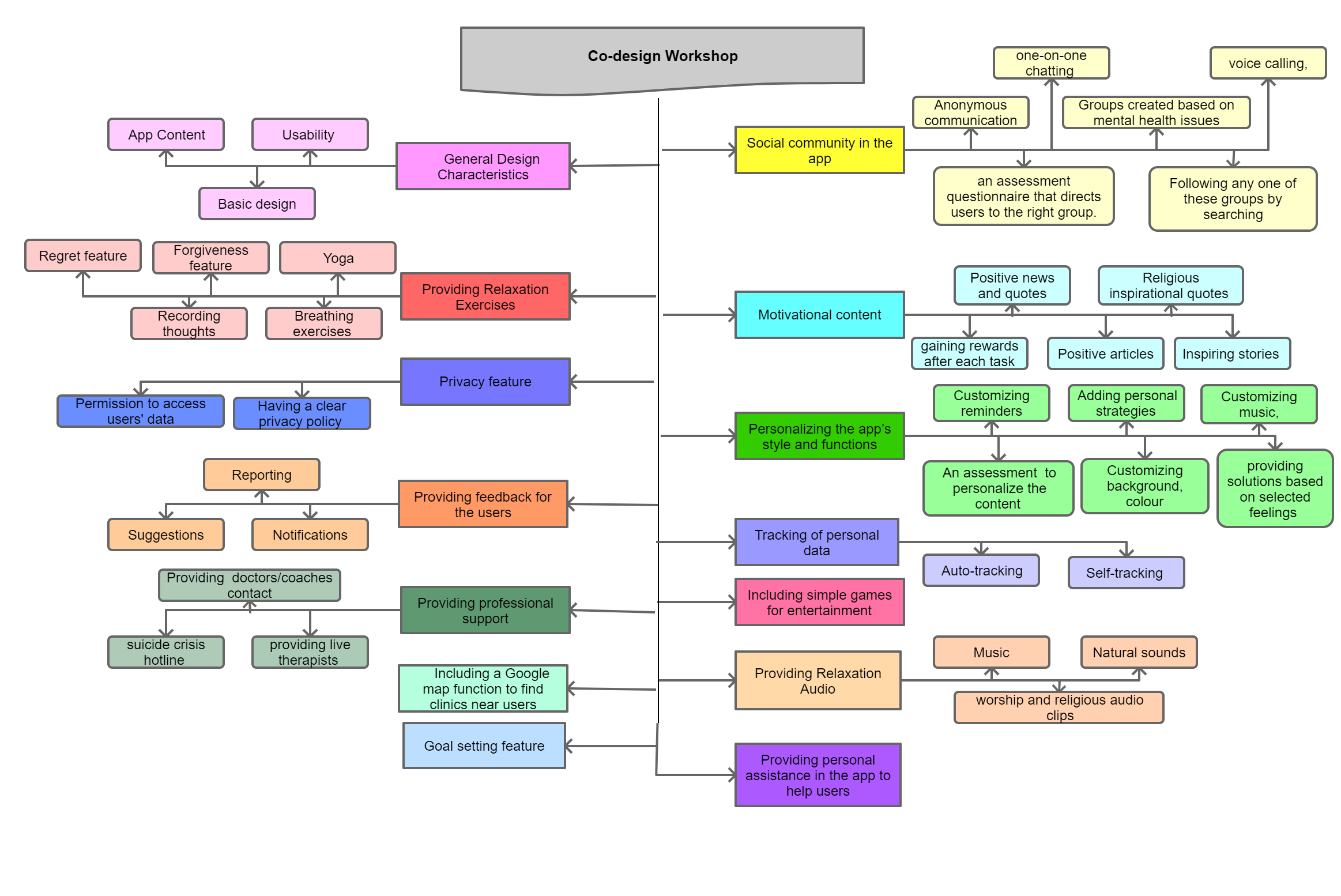


Appendix: Final Themes of Phase 3

Supplement: Multimedia Appendix 1 [file formative_v5i2e18172_app1.docx]
